# Serial Femoral Fractures in An Amputation Stump: A Case Report

**DOI:** 10.2174/1874325001711010316

**Published:** 2017-04-20

**Authors:** M. Nannaparaju, K. Annavaram, R. Anwar, W. S. Khan, J. Hambidge

**Affiliations:** 1 Havering and Redbridge University Hospitals NHS Trust, Romford, Essex RM7 0AG, London, United Kingdom; 2 Princess Alexandra Hospital, Hamstel Road, Harlow CM20 1QX, London, United Kingdom; 3 University College London Institute of Orthopaedics and Musculoskeletal Science, Royal National Orthopaedic Hospital, Stanmore HA7 4LP, London, United Kingdom

**Keywords:** Above-knee amputation, Fracture, Dynamic hip screw, Open reduction and internal fixation, Complications, Rehabilitation

## Abstract

We report a case of an above-knee amputee who underwent dynamic hip screw fixation for a proximal femoral fracture and then open reduction and internal fixation a few years later for a further femoral fracture in the same stump. The patient had a good outcome. We aim to discuss the challenges in decision making, surgical technique and potential complications for the patient.

## 

Isolated femoral fractures in above-knee amputees have been described in the literature [1, 2], and the management of these injuries presents a number of challenges to the surgeon in view of altered anatomy. These include difficulty in the diagnosis, immobilization, surgical reduction and stabilization, as well as post-operative rehabilitation. The goal of treatment is the return of the amputee to their pre-fracture status that includes satisfactory and functional use of a prosthetic limb.

Although isolated femoral fractures in amputees have been reported before there are no reports describing serial femoral fractures requiring operative treatment in the same amputation stump. We report a case of an above-knee amputee who underwent dynamic hip screw fixation for a proximal femoral fracture and then open reduction and internal fixation a few years later for a further femoral fracture in the same stump. We aim to discuss the challenges in decision making and surgical technique, potential complications and long term implications for the patient.

## CASE REPORT

**Fig. (1):**
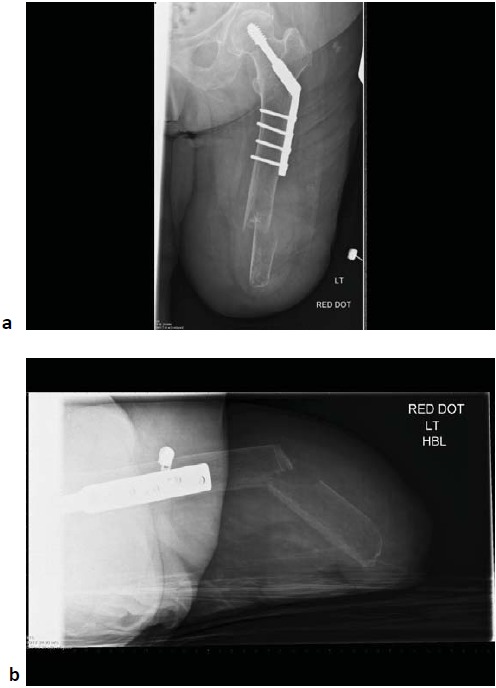
Antero-posterior (a) and lateral (b) radiograph on admission showing a distal femoral shaft fracture with a previous dynamic hip screw fixation.

A 75 year old above-knee amputee who mobilized with an above-knee prosthesis presented to the emergency department with sudden onset of pain in her left thigh following a fall whilst getting out of bed. Weight bearing was not possible due to pain. She had undergone an above knee amputation as a life-saving procedure, ten years prior following necrotizing fasciitis and severe sepsis, possibly triggered by cellulitis in the ipsilateral lower leg. She rehabilitated successfully following the index procedure with a prosthetic limb and returned to her normal activities that included driving. Around five years following her index procedure, she had a fall whilst trying to stand up from a seated position, and sustained a left sided extra capsular neck of femur fracture. The fracture was stabilized with dynamic hip screw (DHS) fixation, and she had a good outcome. She had no other medical or surgical history of note. On examination, there was a deformity in the distal half of the stump that was associated with significant local bony tenderness, swelling and bruising. A lateral well healed surgical scar was noted in the proximal half of the stump. The overlying skin was intact and she had normal peripheral sensation and circulation. No other injuries were identified. Radiographs (Fig. **1**) showed an osteopaenic femur with a fracture of the distal femur almost 8 cm proximal to the amputation stump. There was a healed proximal femoral fracture with an in situ DHS fixation.

**Fig. (2):**
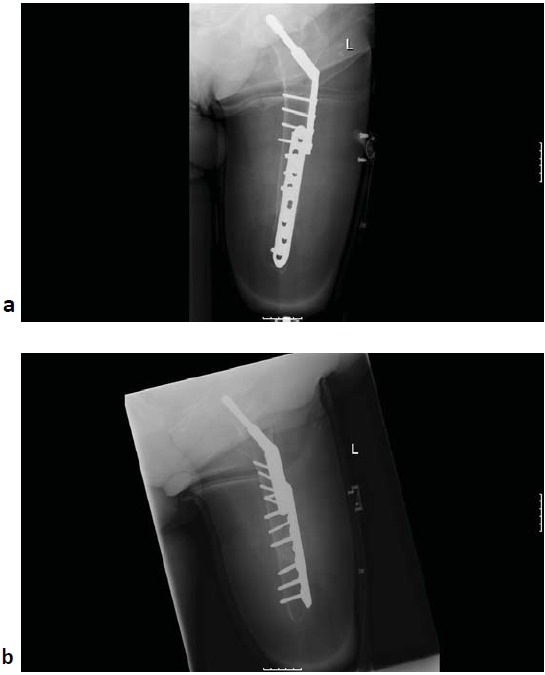
Antero-posterior (a) and lateral (b) radiograph following open reduction and internal fixation of the distal femoral fracture.

Following informed consent, surgical fixation was deemed the most appropriate management. During informed consent the potential benefits of surgery were considered including the maintenance of length that would increase the chances of returning to an earlier level of activity, and the reduced chances of needing the socket revised. With the patient supine, the previous lateral proximal femur incision was extended down to join the stump crease. Superficial tissues were dissected and fascia lata incised to the appropriate length. Vastus lateralis muscle was reflected anteriorly and retracted together with the quadriceps. The fracture was reduced and an eight-hole combi dynamic compression plate was used to stablise it. The plate was placed anteriorly on the shaft and the length was appropriate to overlap with the previous lateral DHS plate to prevent any stress riser. Due to the poor bone quality, combination of non-locking and locking screws was used to fix the construct (Fig. **2**). Cancellous bone chips were used at the fracture site as graft. Good reduction of the fracture was achieved. The fascia lata and skin were closed satisfactorily.

**Fig. (3):**
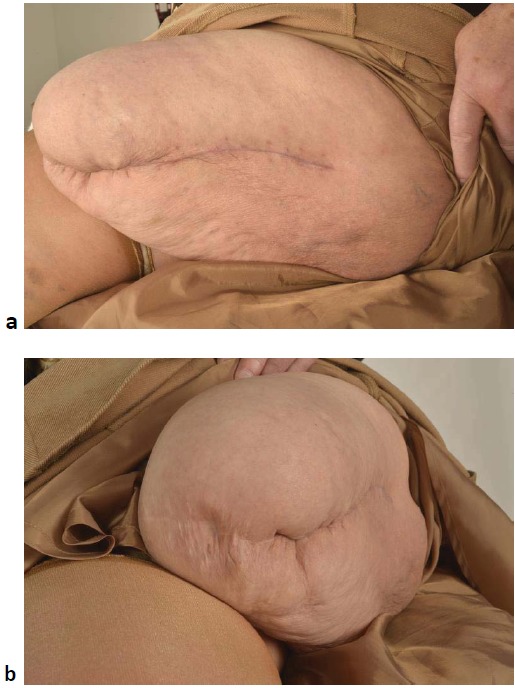
Well healed lateral (a) and stump (b) scar six months post-operatively.

Post-operatively, she had a déjà vu of phantom pain that was significant but resolved with pain team input. The patient started using her prosthetic limb six weeks after the procedure without any difficulty, and was back to her pre-operative mobility at three months. She returned to her normal activities which included driving, fairly soon. At the most recent follow up at six months, there was no evidence of soft tissue infection or wound dehiscence (Fig. **3 **).

## DISCUSSION

Fractures of the residual limb in an amputee are not uncommon. An extensive review of literature yielded 161 in 151 patients [1, 2]. Bowker *et al.* [1] in their series of ninety cases highlighted the epidemiological data and recommendation for the management of this complication. The combination of old age, stump bone osteoporosis and muscle atrophy increases the risk of a stump fracture in an amputee [3], and minimal trauma can lead to a stump fracture. According to Freese [4], a fracture in an amputated leg behaves like a fracture in a normal extremity, and that osteoporosis in the stump had no negative effect on callus formation. In 1949, Slocum [5] condemned open reduction and internal fixation within the portion of the residual limb covered by prosthesis, stating that neither the scar nor metal device would be tolerated well. In 1981, Lewallen and Johnson observed that four out of nine cases treated with internal fixation had complications. They advised conservative management if possible, because of the relatively rapid rate of union of the fracture without internal fixation. A review of the literature suggests that most of the amputees with fracture could be treated conservatively provided the methods used avoided malunion and intraarticular complications.

Bowker *et al.* [1] and other authors [2] felt that properly closed operative wounds did not lead to troublesome scars in amputees. They added that easily workable plastic in modern prosthesis and fixation devices presented a minimum problem after internal fixation. The majority of these cases involved either a fracture of the proximal femur or of the shaft. To date there is no reported case of sequential fractures in the same stump and hence, no guidance regarding treatment is available. In our case, the decision for operative management was made after discussion with the patient who was highly motivated and independent with good mobility. Besides poor bone quality, a major challenge was the choice of fixation devices. One option available to us was to change the previous DHS plate to a longer plate but this would have required a very long plate. Another option was to use a compression plate laterally but this could have created a stress riser between the two plates leading to a peri-prosthetic fracture. We therefore decided to fix the fracture using a plate anteriorly that had sufficient length to overlap the previous DHS plate. This reduced the concentration of stress at the plate-bone interface. Careful consideration was given to the surgical approach and previous lateral incision was extended to fuse with the stump crease. This avoided a new incision in a different plane and also minimized the risk of soft tissue damage and potential complications of infection and skin dehiscence.

Another important consideration in this case, and with all amputees is the maintenance of length. Stump length has been shown to have a significant impact on physiological cost index and comfort walking speed in transfemoral amputees [6]. This was a consideration that was weighted in the risk benefit assessment during the informed consent process in this patient.

Phantom limb pain is common in fresh amputees. This sensation resembles somatosensory events experienced in the limb before amputation and is predominantly a replica of distressing pre-amputation lesions and pains experienced at or near the time of amputation. Reports of ‘somatosensory memories’ are less common when there has been a discontinuity, or a pain-free interval, between the experience of pain and amputation [7]. There is little information in the literature about phantom pain in amputees with subsequent fractures. Interestingly our patient had a déjà vu of phantom limb pain after fixation. Fortunately, this gradually subsided after few weeks with pain medication.

## CONCLUSION

We conclude that careful planning is required in order to manage complex stump fractures. A detailed discussion about the various treatment options is needed, and realistic aims need to be set after discussion with the patient. Biomechanical considerations, including osteopenia and stress risers, should guide the choice of fixation and the patient must be warned about the recurrence of phantom pain.
